# Adropin, S100A1, and SERCA2b Dysregulation in Coronary Artery Disease: Molecular and In Silico Insights into Calcium Signaling and Metabolic Dysfunction

**DOI:** 10.3390/biomedicines14071430

**Published:** 2026-06-24

**Authors:** Onur Aslan, Harika Topal Önal, Meral Urhan Küçük, Emre Dirican

**Affiliations:** 1 Department of Cardiology, Tarsus State Hospital, 33460 Mersin, Turkey; 2 Medical Laboratory Techniques, Vocational School of Health Services, Toros University, 33140 Mersin, Turkey; 3 Department of Medical Biology, Faculty of Medicine, Mustafa Kemal University, 31060 Hatay, Turkey; meralurhan@hotmail.com; 4 Department of Biostatistics, Faculty of Medicine, Mustafa Kemal University, 31060 Hatay, Turkey; emredirican@mku.edu.tr

**Keywords:** adropin, S100A1, SERCA2b, coronary artery disease, type 2 diabetes mellitus

## Abstract

**Background/Objectives:** Coronary artery disease (CAD) is a leading cause of cardiovascular morbidity and mortality worldwide. Type 2 diabetes mellitus (T2DM) further increases CAD risk through metabolic disturbances and endothelial dysfunction. Adropin, S100A1, and SERCA2b are important regulators of endothelial function, energy metabolism, and calcium homeostasis. This study aimed to investigate the gene and protein expression levels of these biomarkers in CAD patients with and without T2DM. **Methods:** Gene and protein expression levels of adropin (ENHO), S100A1, and SERCA2b were evaluated in peripheral blood samples obtained from healthy controls (*n* = 50), CAD patients (*n* = 46), and CAD patients with T2DM (CAD+T2DM) (*n* = 40). Gene expression was determined using real-time PCR, while protein levels were measured with ELISA. Additionally, in silico bioinformatics analyses, such as protein–protein interaction networks and pathway enrichment analyses, were performed to explore potential molecular relationships among these biomarkers. **Results:** Adropin and ENHO gene expression levels were significantly lower in CAD patients and inversely related to the SYNTAX score. S100A1 levels were also reduced, and SERCA2b gene expression was significantly decreased, especially in the CAD+T2DM group. Bioinformatics analyses revealed that these molecules participate in interconnected pathways related to calcium signaling, cardiac muscle contraction, and metabolic regulation. **Conclusions:** These findings demonstrate links between altered levels of adropin, S100A1, and SERCA2b and CAD with or without T2DM. However, these observations are preliminary and need validation in larger prospective studies and mechanistic research before drawing definitive conclusions about their clinical utility, disease progression, or prognostic value.

## 1. Introduction

Coronary artery disease (CAD) is a serious cardiovascular condition caused by the narrowing or blockages in the coronary arteries that supply the heart, due to atherosclerotic plaques [1]. Because of their dynamic physiology, coronary arteries are the main vessels delivering oxygen and nutrients to the heart muscle, and plaque buildup in these arteries can cause myocardial ischemia or a myocardial infarction [2]. There is a strong bidirectional relationship between Type 2 diabetes (T2DM) and CAD because of its influence on the atherosclerotic process [3]. Metabolic disorders such as hyperglycemia, insulin resistance, and systemic inflammation seen in diabetic individuals accelerate the development of atherosclerosis and greatly increase the risk of CAD [4]. Managing blood glucose levels and controlling other cardiovascular risk factors are essential for lowering the risk of CAD [1].

CAD continues to be a leading cause of morbidity and mortality worldwide. According to the World Health Organization (WHO), cardiovascular diseases cause about 17.9 million deaths each year, making up nearly 32% of all global deaths, with ischemic heart disease accounting for the largest share of these deaths [5]. Data from the Global Burden of Disease (GBD) Study also show that ischemic heart disease remains the top cause of disability-adjusted life years (DALYs) and early death globally, creating a significant healthcare and socioeconomic burden [6]. The increasing prevalence of type 2 diabetes mellitus, obesity, and an aging population further increase the incidence and complexity of CAD, emphasizing the need for better molecular biomarkers for early diagnosis, risk stratification, and disease monitoring [7].

Because of its complex pathophysiological processes, including many risk factor-related and endothelium-related factors, CAD is a multifaceted disease influenced not only by metabolic disorders but also by genetic predisposition and environmental factors [8,9,10]. Understanding the molecular mechanisms underlying CAD, along with well-known atherosclerotic cardiovascular risk factors, and identifying potential biomarkers could serve as important guides for diagnosis and treatment. In this study, both the expression and protein levels of adropin (ENHO), S100A1, and SERCA2b genes were evaluated in individuals diagnosed with CAD alone and in those with type 2 diabetes accompanying CAD. The data obtained were compared with healthy individuals, and the potential biomarker value of these genes related to CAD was investigated.

Adropin is a peptide encoded by the ENHO gene involved in metabolic processes such as energy homeostasis, endothelial function, and insulin sensitivity [11,12,13]. It has been reported that adropin is expressed in the liver, brain, and endothelial cells, and that low adropin levels are associated with cardiometabolic diseases [14,15]. Additionally, adropin has been shown to support vascular functions by increasing nitric oxide synthesis, which also plays an important role in cardiovascular pathophysiology [16]. However, there are very limited studies examining the direct relationship between adropin, ENHO gene expression, and CAD.

S100A1 is a protein present in cardiac muscle cells that plays a key role in intracellular calcium signaling [17]. Calcium ions are fundamental molecules that regulate the contraction and relaxation cycle of cardiomyocytes and are also crucial in the event of myocardial damage. It has been suggested that the S100A1 protein improves myocardial function by optimizing calcium balance and offers protective effects in conditions such as heart failure or left ventricular wall motion defect [18]. The relationship between this protein, which may be strongly linked to myocardial damage and contraction regarding its function and effect, and CAD has not yet been fully clarified.

The sarcoplasmic reticulum calcium ATPase (SERCA2b) gene encodes SERCA2b, a calcium transport protein in heart muscle cells. This protein is essential for pumping calcium back into the cell, helping regulate the contraction and relaxation cycle of heart muscle cells [19]. Considering calcium’s role in myocardial contractility and damage, this key function of SERCA2b in heart performance may provide valuable insights into the potential link between heart health and the risk of coronary artery disease (CAD). Studies suggest that the effects of the SERCA2b gene on calcium regulation in heart muscle could contribute to the development of cardiovascular diseases [20,21,22]. However, data regarding the relationship between the SERCA2b gene and CAD remain limited. SERCA2b is one of the major isoforms encoded by the ATP2A2 gene. In this study, we focused specifically on the SERCA2b isoform because of its well-known role in calcium homeostasis and its potential involvement in cardiometabolic disorders. References to ATP2A2 in the bioinformatic analyses pertain to the parent gene encoding SERCA2 isoforms.

Considering myocardial nutrition, wall motion, and calcium-based electrolyte balance in coronary artery disease, it is natural that the interactions among these three genes are of interest. While S100A1 facilitates cardiac muscle relaxation by modulating SERCA2b activity, adropin may indirectly influence these systems through energy balance and insulin signaling. Therefore, changes in the expression levels of these molecules are strong candidates for guiding our understanding of the pathophysiology and outcomes of cardiometabolic diseases. Although ENHO/adropin, S100A1, and SERCA2b have traditionally been investigated separately, emerging evidence suggests that these molecules are functionally interconnected through pathways involved in energy metabolism, endothelial function, calcium homeostasis, and myocardial contractility. Adropin primarily regulates metabolic balance and endothelial health, whereas S100A1 and SERCA2b are key modulators of intracellular calcium cycling and cardiac muscle performance. Since metabolic dysregulation, endothelial dysfunction, and impaired calcium handling are central mechanisms in both CAD and T2DM, evaluating these biomarkers together may provide a more comprehensive understanding of the molecular changes underlying cardiometabolic disease.

This study aimed to analyze gene expression and serum protein levels of adropin, S100A1, and SERCA2b in individuals with coronary artery disease (CAD) and CAD accompanied by type 2 diabetes mellitus. The primary goal was not only to assess the diagnostic relevance of these biomarkers individually but also to investigate whether they collectively form an interconnected molecular network involved in endothelial dysfunction, calcium homeostasis, and metabolic dysregulation. By combining experimental expression analyses with bioinformatic pathway exploration, we aimed to provide a broader mechanistic framework linking these molecules to CAD pathophysiology, disease severity, and potential clinical applications.

## 2. Material and Methods

### 2.1. Ethical Statement

This prospective cross-sectional study was conducted in accordance with the human subjects study protocol of the Declaration of Helsinki, after receiving ethics committee approval numbered 03.05.2024/7. The study included a total of 136 volunteer participants: 46 individuals diagnosed with CAD, 40 individuals diagnosed with CAD and Type 2 diabetes, and 50 healthy individuals without any chronic diseases. These participants applied to the cardiology outpatient clinic of a secondary care state hospital between April 2024 and October 2024. The total number of participants was determined through power analysis using G-power v.3.1.9.6. The exclusion criteria included individuals under 18 years of age, pregnant or breastfeeding women, patients with acute coronary syndrome, patients with chronic inflammatory disease, chronic kidney disease, acute or chronic infection, heart failure with low ejection fraction, patients who have been hospitalized or admitted to the emergency department due to heart failure within the past 3 months, and patients with health problems such as cancer or metabolic syndrome.

### 2.2. Analysis of Clinical Parameters

The demographic characteristics (age, gender) and physical and clinical symptoms of the volunteers in the study were recorded by a cardiologist. Standard laboratory tests were performed on peripheral venous blood samples, including total cholesterol (TT), triglycerides (TG), low-density lipoprotein cholesterol (LDL), high-density lipoprotein cholesterol (HDL), glucose (Glu), creatinine (Cre), and hemoglobin (Hb).

### 2.3. Measurement of Serum Adropin, S100A1 and SERCA2b Levels

Plasma samples collected from the peripheral blood of volunteers were analyzed using the Enzyme-linked Immunosorbent Assay (ELISA) method with BT Lab kits (BT Lab, Shanghai, China; Cat no: E3231Hu for adropin, E4718Hu for S100A1, and E7673Hu for SERCA2b). Protein levels were measured following the protocols provided with the BT Lab ELISA kits. To determine protein levels, the color change resulting from the reactions was detected at wavelengths between 450 and 520 nm using an ELISA microplate reader (Multiskan FC, Thermo Fisher Scientific, Vantaa, Finland). Protein concentrations were calculated based on the absorbance differences between these two wavelengths.

### 2.4. Gene Expression Analyses

In this study, after cDNA was synthesized from RNAs isolated from volunteers’ peripheral blood samples, adropin, S100A1, and SERCA2b mRNA levels were analyzed using real-time PCR. qPCR conditions included a single-step cycle at 95 °C for 2 min, followed by 40 cycles of 10 s at 95 °C and 60 s at 56 °C, with a final melting curve analysis between 60 and 95 °C. After monitoring the melting curves, amplicons specific to the target genes were confirmed. The 2^−ΔΔCT^ method was used to calculate ENHO gene expression levels.

### 2.5. Total RNA Isolation

Total RNA was isolated from peripheral blood samples obtained from volunteers using the kit (InnuScreen RNA Mini Kit, Analytik Jena GmbH, Jena, Germany, Cat no: 845-KS-2010250). The kit’s protocol included RNeasy Mini Spin Columns, 1.5 mL and 2 mL collection tubes, Buffer RLT (45 mL), Buffer RW1 (45 mL), Buffer RPE (11 mL), and RNase-Free Water (10 mL).

RNA concentration and purity were measured using a NanoDrop spectrophotometer (Thermo Fisher Scientific, Wilmington, DE, USA) before cDNA synthesis. Samples with A260/A280 ratios between 1.8 and 2.1 were considered suitable for subsequent molecular analyses. No biological samples were excluded after RNA quality assessment, as all samples met the established quality-control standards.

### 2.6. cDNA Synthesis

cDNA was synthesized from the total RNA samples collected using the Thermo kit (Applied Biosystems, Thermo Fisher Scientific, Foster City, CA, USA; Cat no: 4368814). The kit components included 10× RT Buffer (1 mL), 10× RT Random Primers (1 mL), 25× dNTP Mix (100 mM, 0.2 mL), and MultiScribe^®^ Reverse Transcriptase (Applied Biosystems, Thermo Fisher Scientific, Foster City, CA, USA)

### 2.7. qRT-PCR

For determining gene expression levels, cDNA samples obtained with the Sigma kit (Sigma-Aldrich, St. Louis, MO, USA) were used. Primer sequences specific to the ENHO, S100A1, and SERCA2b genes, along with GAPDH as a housekeeping gene, were employed for gene expression analysis (Table 1). GAPDH was used as the endogenous reference gene for normalization. Relative gene expression levels were calculated using the 2^−ΔΔCt^ method.

### 2.8. Collection and Calculation of Patients’ SYNTAX Values

The patients’ SYNTAX scores were independently assessed by two experienced cardiologists through a retrospective review of coronary angiography images. In cases of discrepancies, a joint decision was reached. Lesions were identified by considering vessels with a diameter of ≥1.5 mm, and factors such as occlusion percentage, vessel diameter, lesion length, thrombus presence, and calcification were analyzed. The total SYNTAX score was calculated using the SYNTAX Score Calculator, and patients were categorized into risk groups: 0–22 (low), 23–32 (medium), and over 32 (high).

### 2.9. In Silico Bioinformatics Analysis

To further explore the molecular connections between ENHO, S100A1, and SERCA2b genes, in silico bioinformatics analyses were performed. Protein–protein interaction (PPI) networks were analyzed using the STRING database (version 12.0). The interaction network was created with a high-confidence interaction score threshold of 0.7.

Functional enrichment analyses were performed using the Enrichr database to identify Gene Ontology (GO) biological processes, molecular functions, and cellular components associated with the studied genes. Additionally, Kyoto Encyclopedia of Genes and Genomes (KEGG) pathway analysis was performed to identify signaling pathways potentially involved in the pathophysiology of coronary artery disease.

To support experimental findings, tissue-specific expression profiles of ENHO, S100A1, and SERCA2b were examined using the Human Protein Atlas database. These analyses aimed to better understand the functional connections of these genes within cardiovascular signaling pathways and calcium regulation mechanisms. Gene Set Enrichment Analysis (GSEA) was not performed in the present study, as the bioinformatic investigation was limited to a predefined set of candidate genes and focused on STRING-based protein–protein interaction and Enrichr-based functional enrichment analyses.

### 2.10. Statistical Analyses

In our study, data were analyzed using SPSS 27 (IBM Corp., Armonk, NY, USA) software. Descriptive statistics included the mean, standard deviation, median (Q1–Q3), frequency, and percentage. The Chi-square test was used for analyzing categorical data, and the Kruskal–Wallis test was applied to continuous variables. For significant results, pairwise comparisons were performed with Bonferroni correction (0.05/3) using the Mann–Whitney U test. Spearman’s Rank correlation examined relationships between continuous variables. Except for pairwise comparisons, an alpha level of 0.05 was set for all tests.

## 3. Results

A total of 136 individuals participated in this study. Participants were divided into three groups: the control group (*n* = 50; 36.8%), the group with a diagnosis of coronary artery disease (CAD) only (*n* = 46; 33.8%), and the group with both CAD and Type 2 diabetes (CAD+Type 2) (*n* = 40; 29.4%). Demographic information, along with clinical and laboratory data, are provided in Table 2.

No statistically significant differences were found between the groups in demographic and lifestyle variables such as age, gender, daily exercise, smoking, and alcohol use among the study participants (*p* > 0.05). In this context, the age distribution was similar, with a median of 58 (52–65) in the control group, 62 (54–70) in the CAD group, and 60.5 (55.5–67) in the CAD+Type 2 diabetes group (*p* = 0.256).

Gender distribution, exercise habits, smoking, and alcohol consumption did not differ significantly between the groups (*p* = 0.357, *p* = 0.224, *p* = 0.192, and *p* = 0.371, respectively) (Table 2). When examining biochemical parameters, glucose (Glu) levels showed significant differences between the groups (*p* < 0.001). Glucose levels were notably higher in the CAD+Type 2 diabetes group [median 140 (110–162)] compared to both the control group [97 (89–109); *p* < 0.001] and the CAD-only group [97.5 (91–113); *p* < 0.001] (Table 2).

There was also a significant difference in LDL cholesterol levels (*p* < 0.001). The LDL levels of individuals in the CAD+Type 2 group [85 (68–113.5)] were significantly lower than those in the control group [122.8 (98–144); *p* < 0.001] and the CAD group [129.5 (97–155); *p* < 0.001] (Table 2). This difference is likely due to the intensive lipid-lowering treatment received by individuals in this group. Aside from this, no statistically significant differences were observed among the groups regarding creatinine, hemoglobin, total cholesterol (TC), HDL, or triglyceride (TT) levels (*p* = 0.096, *p* = 0.064, *p* = 0.178, *p* = 0.231, and *p* = 0.421, respectively) (Table 2).

### 3.1. Serum Adropin, S100A1 and SERCA2b Levels and Gene Expression Levels

This study compares serum levels and gene expressions among control, CAD, and CAD+T2DM groups. All variables are presented as medians and interquartile ranges (Q1–Q3).

Adropin levels were measured as 170.6 ng/mL (119.9–268.6) in the control group, 81.3 (59–128.3) in the CAD group, and 63.1 (54.2–76.7) in the CAD+T2DM group. Adropin levels were found to be significantly lower in the patient groups (*p* < 0.001). This suggests that adropin deficiency may be associated with cardiometabolic diseases. It was observed that adropin levels decreased as the SYNTAX score increased.

S100A1 protein had the highest level in the control group [262.9 (200.2–332.8)], followed by 141.6 (120.2–199.8) in the CAD group and 126.8 (117.1–152.5) in the CAD+T2DM group (*p* < 0.001). This finding indicates that S100A1 levels in myocardial cells decrease as the disease progresses. Additionally, a strong correlation was observed between the increase in SYNTAX score and the decrease in S100A1, and this relationship was independent of all other factors. SERCA2b levels were 5.8 (4.6–7.6) in the control group, 4.9 (3.3–6.1) in the CAD group, and 3.7 (3.3–4.5) in the CAD+Type 2 group, with differences between groups being statistically significant (*p* < 0.001).

According to gene expression analysis, Enho 2^−∆∆CT^ levels showed a borderline significant difference between groups (*p* = 0.049). It was 0.9 (0.7–1.5) in the control group, 0.8 (0.5–1.1) in the CAD group, and 0.8 (0.6–1.7) in the CAD+T2DM group (Table 3).

The S100A1 2^−∆∆CT^ gene expression level was measured as 1.0 (0.2–3.6) in the control group and 0.05 (0.02–0.08) and 0.05 (0.01–0.12) in the CAD and CAD+T2DM groups (*p* < 0.001) (Table 3). This indicates that S100A1 is also downregulated at the genetic level.

SERCA2b 2^−∆∆CT^ level was 0.2 in both the control and CAD groups, whereas it was 0.8 in the CAD+T2DM group, showing a sharp decrease to 0.2 (0.1–0.8). This reduction made the difference between the groups statistically significant (*p* = 0.008) (Table 3). It is suggested that the main factor is the presence of diabetes. In the patient groups, the SYNTAX score was 18 (16–23) in the CAD group and 19 (14–20) in the CAD+T2DM group, with no statistically significant difference between them (*p* = 0.089) (Table 3).

### 3.2. Correlation Findings

In the control group, a strong and statistically significant positive correlation was observed between adropin and SERCA2b levels (r = 0.768, *p* < 0.01). Likewise, significant positive correlations were found between adropin and S100A1 (r = 0.596, *p* < 0.01), as well as between SERCA2b and S100A1 (r = 0.581, *p* < 0.01). No significant relationships were detected between glucose levels and these target proteins (*p* > 0.05).

In the group with coronary artery disease (CAD), adropin and SERCA2b levels remained strongly and positively correlated (r = 0.773, *p* < 0.01). Similarly, adropin showed a significant positive correlation with S100A1 (r = 0.760, *p* < 0.01), and SERCA2b with S100A1 (r = 0.869, *p* < 0.01). While glucose levels did not significantly correlate with any of the studied markers, the SYNTAX score demonstrated strong inverse correlations with adropin (r = −0.756, *p* < 0.01), SERCA2b (r = −0.774, *p* < 0.01), and S100A1 (r = −0.801, *p* < 0.01) (Table 4).

In the CAD group with concomitant type 2 diabetes, a significant positive correlation was also observed between adropin and SERCA2b levels (r = 0.704, *p* < 0.01). Additionally, adropin levels showed a positive correlation with S100A1 (r = 0.640, *p* < 0.01), and SERCA2b with S100A1 (r = 0.793, *p* < 0.01). In contrast, glucose levels did not display statistically significant correlations with any of the biomarkers. The SYNTAX score, however, was negatively and significantly correlated with adropin (r = −0.665, *p* < 0.01), SERCA2b (r = −0.724, *p* < 0.01), and S100A1 (r = −0.737, *p* < 0.01) (Table 4).

### 3.3. Molecular Findings Related to Adropin, S100A1 and SERCA2b Levels

**Figure 1:**
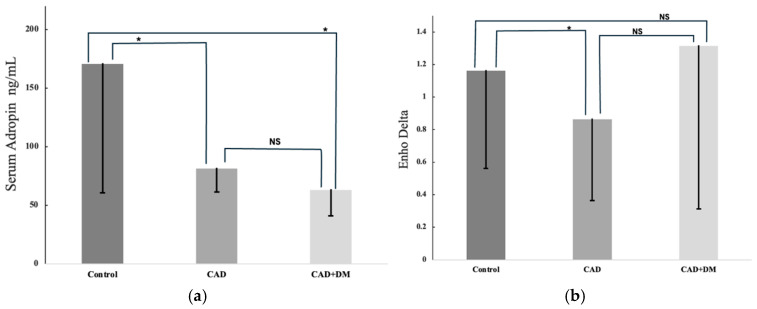
Comparison of adropin Levels and ENHO Gene Expression Among Study Groups. (**a**) Serum adropin levels in the control, coronary artery disease (CAD), and CAD with type 2 diabetes (CAD+DM) groups. Adropin levels were significantly higher in the control group compared to both CAD and CAD+DM groups (* *p* < 0.05). No significant difference was observed between the CAD and CAD+DM groups (NS). (**b**) Comparison of 2^−ΔΔCT^ values representing ENHO gene expression among the groups. ENHO expression was significantly higher in the control group compared to the CAD group (* *p* < 0.05), while no significant differences were found between the CAD and CAD+DM groups or between the control and CAD+DM groups (NS).

Serum adropin levels were compared among the control, CAD, and CAD+T2DM groups (Figure 1a). Adropin levels were significantly higher in the control group and showed a statistically significant difference compared to both the CAD and CAD+T2DM groups (*p* < 0.05). However, no significant difference was observed between the CAD and CAD+T2DM groups (NS). This finding suggests that adropin levels are mainly decreased by coronary artery disease, and the presence of type 2 diabetes does not have an additional effect. The fact that adropin levels show a clear correlation with the increase in SYNTAX score highlights the importance of this result.

**Figure 2:**
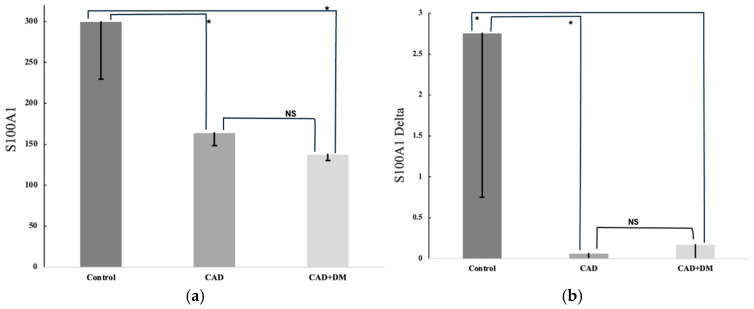
Comparison of S100A1 Protein Levels and S100A1 Gene Expression Among Study Groups. (**a**) Serum S100A1 protein levels across the control, coronary artery disease (CAD), and CAD with type 2 diabetes (CAD+DM) groups. S100A1 concentrations were significantly higher in the control group compared to both the CAD and CAD+DM groups (* *p* < 0.05). No significant difference was observed between the CAD and CAD+DM groups (NS). (**b**) 2^−ΔΔCT^ values representing S100A1 gene expression were compared between groups. The control group exhibited markedly higher S100A1 expression compared to both CAD and CAD+DM groups (* *p* < 0.05). However, the difference between the CAD and CAD+DM groups was not statistically significant (NS: non-significant).

When adropin levels and ENHO expression are analyzed together, both biomarkers show a similar decreasing trend, indicating that suppression at the genetic level is more closely linked to coronary artery disease (Figure 1b). S100A1 protein levels are significantly higher in the control group and are notably greater compared to both the CAD and CAD+T2DM groups (*p* < 0.05). However, no significant difference was observed between the two patient groups (CAD and CAD+T2DM) (NS) (Figure 2a). S100A1 gene expression (2^−∆∆CT^) levels were markedly higher in the control group, while they decreased significantly in both the CAD and CAD+T2DM groups (*p* < 0.05) (Figure 2b).

**Figure 3:**
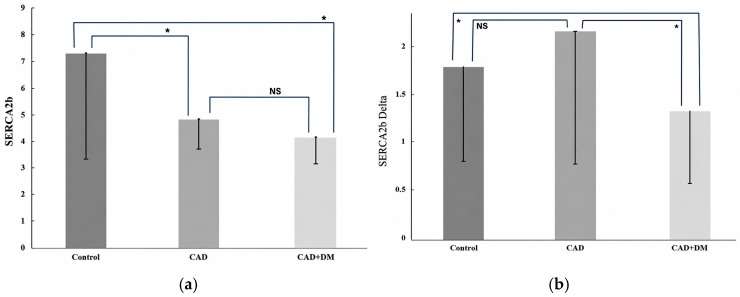
SERCA2b Levels and SERCA2b Gene Expression Across Study Groups. (**a**) Comparison of SERCA2b protein levels among the control, coronary artery disease (CAD), and CAD with type 2 diabetes (CAD+DM) groups. SERCA2b levels were significantly higher in the control group compared to both CAD and CAD+DM groups (* *p* < 0.05). No statistically significant difference was observed between the CAD and CAD+DM groups (NS). (**b**) 2^−ΔΔCT^ values reflecting SERCA2b gene expression were compared across the groups. Expression levels were significantly higher in the CAD group compared to the CAD+DM group (* *p* < 0.05). Although no significant difference was found between the control and CAD groups, SERCA2b expression was significantly elevated in the control group relative to the CAD+DM group (* *p* < 0.05).

When examining the group-based distribution of serum SERCA2b levels, they were significantly higher in the control group compared to both CAD and CAD+T2DM groups (*p* < 0.05) (Figure 3a). No statistically significant difference was found between the CAD and CAD+T2DM groups (NS). This clearly indicates that SERCA2b levels decrease with CAD. The presence of diabetes does not appear to have an additional impact on the results.

More interesting data appears in the comparison of SERCA2b gene expression levels. In the CAD+T2DM group, significantly lower expression was observed compared to both the control and CAD groups (*p* < 0.05), but there was no statistically significant difference between the control and CAD groups (NS) (Figure 3b). This data indicates that SERCA2b gene expression is more notably suppressed when diabetes is present, suggesting that diabetes has an inhibitory effect at the genetic level (Figure 3). At this stage, coronary artery disease does not seem to make a clear difference.

### 3.4. In Silico Functional Analysis of ENHO, S100A1 and SERCA2b

**Figure 4:**
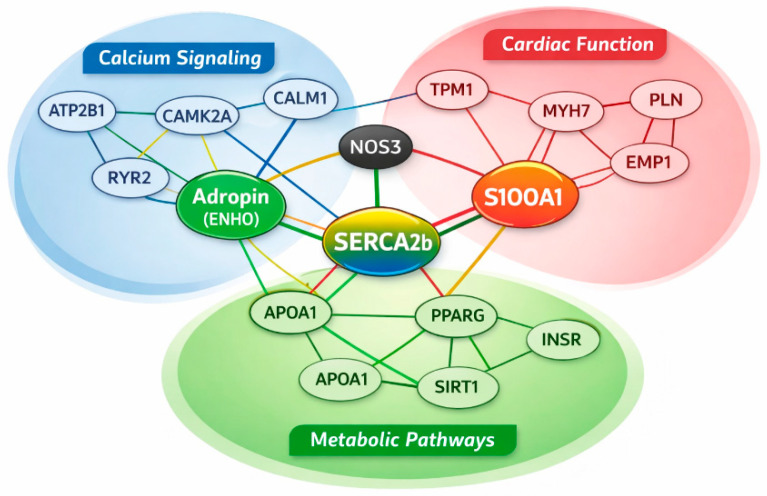
Protein–protein interaction (PPI) network of ENHO, S100A1, and SERCA2b obtained from the STRING database. The network shows functional interactions among calcium signaling regulators, including ATP2A2, RYR2, and phospholamban (PLN), indicating that these biomarkers are involved in interconnected pathways related to myocardial calcium handling and cardiovascular regulation.

To further investigate the potential molecular relationships among the studied biomarkers, an in silico protein–protein interaction (PPI) analysis was performed using the STRING database. The interaction network revealed functional associations between ENHO, S100A1, and SERCA2b, along with several proteins involved in cardiac calcium regulation (Figure 4). The analysis identified functional interactions between S100A1 and key proteins involved in cardiac calcium regulation, including ryanodine receptor 2 (RYR2), phospholamban (PLN), and ATP2A2, which are critical regulators of sarcoplasmic reticulum calcium handling.

Functional enrichment analysis showed that ENHO, S100A1, and SERCA2b mainly participate in biological processes such as calcium ion transport, cardiac muscle contraction, and regulation of endothelial function. Molecular function analysis identified calcium ion binding and ATPase activity as the primary roles of these proteins.

**Figure 5:**
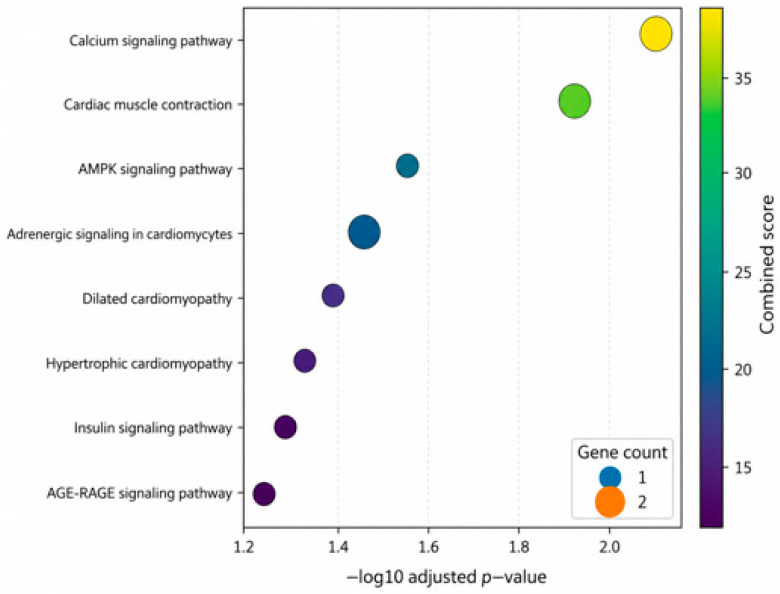
KEGG pathway enrichment analysis of ENHO, S100A1, and ATP2A2/SERCA2b. Dot size represents the number of overlapping genes, whereas color intensity indicates the enrichment score. The *x*-axis shows enrichment significance expressed as −log10 adjusted *p*-value.

KEGG pathway analysis further revealed enrichment in the calcium signaling pathway, cardiac muscle contraction, and the AMPK signaling pathway (Figure 5). These pathways are essential for myocardial contractility, metabolic regulation, and vascular homeostasis, which are known to be key mechanisms in the development of coronary artery disease.

Tissue expression analysis from the Human Protein Atlas database revealed that S100A1 and SERCA2b are highly expressed in cardiac muscle tissues, while ENHO is mainly found in liver, endothelial, and metabolic tissues. These findings support the physiological importance of these biomarkers in cardiovascular metabolism and myocardial function.

## 4. Discussion

In this study, both the expression levels and the corresponding protein levels of adropin, S100A1 (S100A1), and SERCA2b genes were examined in individuals diagnosed with CAD alone and in those diagnosed with Type 2 diabetes accompanying CAD. Our main finding was that adropin and ENHO gene expression were significantly decreased in CAD patients and showed a clear correlation with the severity of CAD. Another key finding was the sharp decrease in S100A1 gene expression and protein levels in CAD patients. A third important finding was the significant impact of the presence of DM on Serca2b gene expression. The coexistence of CAD and DM, rather than CAD alone, appears to be closely related to the expression level of this gene. Beyond their individual biomarker potential, our findings support the concept that adropin, S100A1, and SERCA2b participate in a coordinated biological network connecting metabolic regulation, endothelial integrity, and calcium signaling. This integrated perspective may better explain the multifactorial nature of CAD than evaluation of each marker in isolation.

Adropin levels and ENHO gene expression were found to be significantly lower in both patient groups compared to the control group. Adropin is a peptide directly involved in energy homeostasis, insulin sensitivity, and endothelial functions, expressed in the heart, brain, liver, and coronary endothelial cells, and encoded by the ENHO gene [23,24]. Consistent with our study, many reports in the literature document a significant decrease in serum adropin levels in CAD patients [25,26]. Moreover, studies have shown that low adropin levels are associated with metabolic disorders such as obesity, insulin resistance, and type 2 diabetes [27,28]. Additionally, adropin has been reported to regulate vascular tone and suppress atherosclerotic processes by increasing endothelial nitric oxide synthase (eNOS) activity in endothelial cells [29]. The notable decrease in adropin levels in the CAD and CAD+T2DM groups in our study supports the crucial role of this molecule in vascular dysfunction and atherosclerosis. The similar reduction in ENHO gene expression suggests that this decline originates at the genetic level. To our knowledge, this is the first study to evaluate ENHO gene expression in patients with CAD. Our findings reveal a clear relationship between the severity of CAD and the expression of this protein and gene. This has significant potential to enhance understanding of the molecular basis underlying different CAD courses in patients with similar risk factors. S100A1 is defined as a calcium-binding protein that regulates calcium signaling in cardiomyocytes [14]. In humans, S100A1 gene expression is highest in cardiomyocytes, especially in the left ventricle, as well as in vascular endothelial cells [30]. This protein is primarily localized in the sarcoplasmic reticulum, mitochondria, and myofibrillar segments and plays a role in various regulatory processes within the cardiovascular system [31]. The positive effects of S100A1 on myocardial contractility have been demonstrated in both in vitro and in vivo models [32,33]. Furthermore, this protein exhibits cardioprotective properties that may delay heart failure progression [34]. In our current study, the significant reduction in S100A1 levels—both at the gene expression and serum levels—suggests that this molecule may be linked to CAD progression. An interesting point is that S100A1 deficiency correlates with left ventricular dysfunction, while S100A1 overexpression reduces contractile dysfunction [35]. S100A1 interacts with the sarcoplasmic reticulum calcium pump (SERCA2b) in a calcium-dependent manner, increasing its activity and facilitating calcium reuptake into the sarcoplasmic reticulum [36]. It also decreases calcium leakage during diastole and enhances calcium release during systole via ryanodine receptor 2 (RyR2). These mechanisms may offer protection against cardiac arrhythmias [37]. Although previous studies have suggested that S100A1 deficiency may be associated with impaired myocardial function and heart failure progression [38], the present study did not evaluate heart failure outcomes or longitudinal clinical events. Therefore, any potential role of reduced S100A1 expression in heart failure development remains hypothesis-generating and requires confirmation in future prospective studies.

SERCA2b is an ATPase that pumps calcium into the sarcoplasmic reticulum in cardiomyocytes and plays a crucial role in maintaining the contraction–relaxation cycle [39]. Reduced SERCA2b expression has been identified as a key molecular cause of cardiac dysfunction [40]. In this study, it was observed that SERCA2b gene expression was significantly suppressed, especially in the CAD+T2DM group. This finding indicates an association between the coexistence of type 2 diabetes and lower SERCA2b expression. Previous studies have reported reduced SERCA2b expression in diabetic cardiomyopathy and its association with ventricular filling impairment and diastolic dysfunction [41,42]. Consistent with these observations, our results suggest that altered SERCA2b expression may be involved in the molecular changes observed in CAD patients with T2DM. However, the present study was not designed to determine causality or to directly evaluate calcium homeostasis. The notable decline in this gene’s expression in DM indicates that the primary treatment focus in CAD+DM should not be solely on coronary artery disease from a cardiovascular perspective. The use of agents such as sodium–glucose cotransporter 2 inhibitors (SGLT2i) in DM treatment may cast doubt on the role of genetic factors in explaining the molecular benefits of these agents.

We believe that the significance of our findings has increased, as they demonstrate a clear correlation in the same direction between the relationship of coronary artery disease with these proteins and genes and the SYNTAX score, which measures the prevalence of CAD. Indeed, as the SYNTAX score rises, the noticeable decrease in adropin and S100A1 suggests that this relationship may be strongly associated not only with the presence of CAD but also with its severity [43,44].

The inverse correlations observed between adropin, S100A1, and SYNTAX scores were of moderate to strong magnitude, indicating that these biomarkers may reflect the anatomical burden and severity of coronary artery disease. From a clinical perspective, biomarkers associated with SYNTAX score could have potential utility in identifying patients with more extensive coronary involvement. However, the present study was not designed to evaluate diagnostic performance, prognostic value, or clinical decision thresholds. Therefore, although these findings support the biological and potential clinical relevance of adropin and S100A1, further prospective studies involving ROC analyses, predictive modeling, and long-term cardiovascular outcomes are necessary before these biomarkers can be reliably used for routine risk stratification or clinical decision-making.

Adropin, S100A1, and SERCA2b are biomolecules involved in essential pathways that maintain cardiovascular system homeostasis, despite their distinct cellular roles. adropin regulates endothelial function by increasing nitric oxide (NO) production and supporting vascular relaxation [24]. It also enhances insulin sensitivity and reduces systemic inflammation by affecting energy metabolism [29]. These actions may directly or indirectly influence ion balance and contractility in cardiomyocytes. S100A1 is a key calcium (Ca^2+^) sensor that controls calcium release from the sarcoplasmic reticulum in cardiomyocytes and governs the contraction–relaxation cycle of the heart muscle [45]. This protein directly modulates SERCA2b activity; studies have shown that S100A1 interacts with SERCA2b to promote calcium reuptake into the sarcoplasmic reticulum and speed up myocardial relaxation [46]. SERCA2b, the ATPase responsible for pumping calcium, plays a central role in maintaining diastolic function by facilitating Ca^2+^ reuptake into the sarcoplasmic reticulum after contraction [47]. In this context, it is reasonable to suggest that adropin could indirectly support the functions of S100A1 and SERCA2b by increasing endothelium-derived NO production and metabolic efficiency. The involvement of these biomarkers in similar signaling pathways or their mutual modulation suggests a systemic dysfunction in the pathophysiology of CAD. Therefore, adropin, S100A1, and SERCA2b can be seen as complementary biomarkers reflecting the effects of CAD on both endothelial function and the myocardial contraction–relaxation cycle. The strong positive correlations between adropin, S100A1, and SERCA2b protein levels observed in our analyses are expected. We believe this comprehensive approach offers valuable insight into how multi-biomarker strategies can improve diagnosis and management of complex cardiovascular diseases, especially in the progression toward heart failure, which is the ultimate endpoint.

The in silico analyses conducted in this study further support the biological importance of ENHO, S100A1, and SERCA2b in cardiovascular physiology. The interaction networks and pathway enrichment analyses showed that these molecules are involved in interconnected pathways that regulate calcium balance, heart muscle contraction, and metabolic signaling. These findings support the idea that disruption of these molecular pathways might contribute to the development and progression of coronary artery disease.

More specifically, pathway enrichment analysis revealed significant involvement in calcium signaling, cardiac muscle contraction, and AMPK signaling pathways. Calcium signaling is essential for myocardial contractility, excitation–contraction coupling, and intracellular homeostasis, and its dysregulation has been implicated in ischemic injury and adverse cardiac remodeling. Similarly, the cardiac muscle contraction pathway is directly related to myocardial performance and may indicate changes in cardiac function seen in CAD patients. The enrichment of the AMPK signaling pathway is especially notable because AMPK is a key regulator of cellular energy metabolism and adaptation to ischemic stress. Given the established roles of metabolic dysfunction, endothelial impairment, and altered calcium handling in CAD, these findings further support the biological relevance of the observed changes in ENHO, S100A1, and SERCA2b expression.

In line with these findings, the protein–protein interaction network analysis revealed functional connections between ENHO, S100A1, and several key calcium-regulating proteins, such as ryanodine receptor 2 (RYR2), phospholamban (PLN), and ATP2A2. This network also emphasizes the molecular links between calcium signaling pathways and cardiovascular regulation in coronary artery disease.

### Limitations

This study’s strengths include the concurrent assessment of gene expression and serum protein levels of adropin, S100A1, and SERCA2b. However, several limitations should be recognized. The relatively small sample size and absence of glycemic control parameters, such as diabetes duration and HbA1c levels, may restrict the applicability of the results. Additionally, ejection fraction and long-term clinical outcomes were not assessed, which limits the ability to evaluate the potential influence of these biomarkers on cardiac function over time. Gene expression analyses were performed using peripheral blood samples instead of cardiac tissue; therefore, the observed expression patterns should be viewed as circulating molecular signatures rather than direct measures of myocardial gene expression. Lastly, inflammatory mediators related to the HMGB1/NLRP3 inflammasome pathway, including HMGB1, IL-1β, NLRP3, ASC, and Caspase-1, were not evaluated due to budget constraints. Future research involving cardiac tissue analysis, long-term follow-up, and additional inflammation-related biomarkers could offer more detailed insights into the mechanisms underlying CAD, especially in patients with type 2 diabetes mellitus.

## 5. Conclusions

In this study, serum levels of adropin, S100A1, and SERCA2b proteins, along with the expression levels of the genes encoding these proteins, were compared in individuals diagnosed with coronary artery disease. Our results showed a significant decrease in these three biomarkers in CAD and CAD+T2DM patients. Considering the roles of these three genes in endothelial functions, metabolic regulation, calcium homeostasis, mitochondrial energy production, and their involvement in the cardiac contraction–relaxation cycle, as well as their interactions with each other, it is clear that these proteins play vital roles in the pathophysiology and prognosis of CAD and may serve as potential biomarkers.

In conclusion, these molecules—adropin, S100A1, and SERCA2b—can be used as potential biomarkers for diagnosing, prognosis, and monitoring the treatment of cardiometabolic disorders. These findings should be confirmed through larger sample sizes and tissue-based studies, paving the way for clinical applications in translational medicine. Future larger cohort and tissue-based studies will help more clearly establish the predictive and prognostic value of these biomarkers.

## Figures and Tables

**Table 1:** Primer sequences used in qRT-PCR analysis.

Genes	Primer Sequences
**ENHO**	F: 5′-CTCAACTCAGGCTCAGGACT- 3′ R: 5′-GACAGTGGAGCTGCCTCAAT-3′
**S100A1**	F: 5′- CTTGGCCATCTGTCCAGAAC-3′ R: 5′-CAATGTGGCTGTCTGCTCAA-3′
**SERCA2b**	F: 5′-TCATCTTCCAGATCACACCGCT-3′ R: 5′-TCAAGACCAGAACATATCGC-3′
**GAPDH**	F: 5′-GAGAGACCCTCACTGCTG-3′ R: 5′-GATGGTACATGACAAGGTGC-3′

**Table 2:** Demographic, clinical and laboratory data in groups.

Variables	Control (*n* = 50, % 36.8)	CAD (*n* = 46, % 33.8)	CAD+T2DM (*n* = 40, % 29.4)	*p* Value
**Age** (median (q1–q3))	58 (52–65)	62 (54–70)	60.5 (55.5–67)	0.256
**Gender** (*n*:%)				
Female	17 (34)	18 (39.1)	15 (37.5)	0.357
Male	33 (66)	28 (60.9)	25 (62.5)	
**Daily Exercise** (*n*:%)				
No exercise	18 (36)	11 (23.9)	9 (22.5)	0.224
Light exercise	26 (52)	30 (65.2)	25 (62.5)	
Moderate exercise	6 (12)	5 (10.9)	6 (15)	
Smoking (*n*:%)				
Yes	31 (62)	26 (56.5)	30 (75)	0.192
No	19 (38)	20 (43.5)	10 (25)	
Alcohol (*n*:%)				
Yes	48 (96)	41 (89.1)	36 (90)	0.371
No	2 (4)	5 (10.9)	4 (10)	
**Glu** (median (q1–q3))	97 (89–109)	97.5 (91–113)	140 (110–162)	**<0.001 ^b,c^**
**Kre** (median (q1–q3))	0.8 (0.7–0.9)	0.77 (0.65–0.92)	0.9 (0.8–1.1)	0.096
**Hb** (mean ± sd)	12.6 ± 1.9	13 ± 2.1	13.6 ± 1.7	0.064
**TT** (mean ± sd)	200.7 ± 51.3	199.7 ± 48.9	187.8 ± 49.3	0.421
**HDL** (mean ± sd)	48.1 ± 11.4	45.2 ± 9	47.8 ± 9.7	0.231
**TC** (median (q1–q3))	140 (104–177.5)	157.5 (113–198)	166.5 (110–305)	0.178
**LDL** (median (q1–q3))	122.8 (98–144)	129.5 (97–155)	85 (68–113.5)	**<0.001 ^b,c^**

For pairwise comparison, ^b^: Control etc CAD+T2DM, ^c^: CAD etc CAD+T2DM.

**Table 3:** Serum adropin, S100A1 and SERCA2b levels, gene expression levels and SYNTAX score distribution.

Variables	Control (*n* = 50)	CAD (*n* = 46)	CAD+T2DM (*n* = 40)	*p*
**Adropin**	170.6 (119.9–268.6)	81.3 (59–128.3)	63.1 (54.2–76.7)	**<0.001**
**S100A1**	262.9 (200.2–332.8)	141.6 (120.2–199.8)	126.8 (117.1–152.5)	**<0.001**
**SERCA2b**	5.8 (4.6–7.6)	4.9 (3.3–6.1)	3.7 (3.3–4.5)	**<0.001**
**Enho 2^−∆∆CT^**	0.9 (0.7–1.5)	0.8 (0.5–1.1)	0.8 (0.6–1.7)	**0.049**
**S100A1 2^−∆∆CT^**	1 (0.2–3.6)	0.05 (0.02–0.08)	0.05 (0.01–0.12)	**<0.001**
**SERCA2b 2^−∆∆CT^**	0.8 (0.2–3.4)	0.8 (0.1–3.1)	0.2 (0.1–0.8)	**0.008**
**SYNTAX**	-	18 (16–23)	16 (14–20)	**0.089**

All variables are expressed as median (interquartile range: Q1–Q3).

**Table 4:** Correlation analyses.

Groups	Variables	Statistics	Glucose (Glu)	Adropin	SERCA2b	S100A1
**Control**	**Glu**	**r**	1	−0.065	−0.053	−0.034
		***p***		0.656	0.72	0.816
	**Adropin**	**r**	−0.065	1	0.768	0.596
		***p***	0.656		<0.001	<0.001
	**SERCA2b**	**r**	−0.053	0.768	1	0.581
		***p***	0.72	<0.001		<0.001
	**S100A1**	**r**	−0.034	0.596	0.581	1
		***p***	0.816	<0.001	<0.001	
**CAD**	**Glu**	**r**	1	−0.244	−0.143	−0.103
		***p***		0.103	0.342	0.496
	**Adropin**	**r**	−0.244	1	0.773	0.760
		***p***	0.103		<0.001	<0.001
	**SERCA2b**	**r**	−0.143	0.773	1	0.869
		***p***	0.342	<0.001		<0.001
	**S100A1**	**r**	−0.103	0.760	0.869	1
		***p***	0.496	<0.001	<0.001	
	**SYNTAX**	**r**	−0.104	−0.756	−0.774	−0.801
		***p***	0.493	<0.001	<0.001	<0.001
**CAD+T2DM**	**Glu**	**r**	1	0.119	0.255	0.152
		***p***		0.466	0.112	0.349
	**Adropin**	**r**	0.119	1	0.704	0.640
		***p***	0.466		<0.001	<0.001
	**SERCA2b**	**r**	0.255	0.704	1	0.793
		***p***	0.112	<0.001		<0.001
	**S100A1**	**r**	0.152	0.640	0.793	1
		***p***	0.349	<0.001	<0.001	
	**SYNTAX**	**r**	−0.033	−0.665	−0.724	−0.737
		***p***	0.839	<0.001	<0.001	<0.001

## Data Availability

The original contributions presented in this study are included in the article. Further inquiries can be directed to the corresponding authors.
